# Correction: Towards an affect intensity regulation hypothesis: Systematic review and meta-analyses of the relationship between affective states and alcohol consumption

**DOI:** 10.1371/journal.pone.0318674

**Published:** 2025-01-29

**Authors:** Anna Tovmasyan, Rebecca L. Monk, Derek Heim

In Table 1, there are errors in the correlation coefficients. Please see the correct Table 1 here.

**Table 1:** Characteristics of included studies.

Number	Authors	Year	Country	Method	Sample size(s)	Sample type	Sample gender(s)	Sample age(s)	Positive or negative	Affect measure	Looked at distinct emotions (Yes/No)	Alcohol measure	Effect size (*r*)	Relevant key findings
1	Austin, Notebaert, Wiers, Salemink, & MacLeod [80]	2020	Netherlands	Experimental, ad libitum taste test	39	General population	24 women	M = 24.82, SD = 7.49, range = 18–59	Negative	VAMS [39]	No	Ad libitum (grams of beverage consumed)	*r* = .25	Beer consumption was higher in the negative affect induction condition.
2	Cyders, Zapolski, Combs, Settles, Fillmore, & Smith [81]	2010	USA	Experimental study, ad libitum taste test	33	Undergraduate psychology students	14 women	M = 22.27, SD = 2.36, range not reported	Positive	PANAS [38]	No	Ad libitum (milliliters of beverage consumed)	*r* = .40	Participants drank more in the positive mood than in the neutral mood condition.
3	de Castro [82]	1990	USA	Diary study	96	General population	63 women	M = 32.9, SD not reported, range = 21–54	Both, as a continuum	7-point Likert scale (elated—depressed, anxious—calm scales)	No	Record everything you drink	*r* (elation-depression scale) = -.33; *r* (anxious-calm scale) = -.17	The degree of elation, not depression, was related to the amount of alcohol ingested. On the calm-anxiety scale, people were mostly drinking when they felt ’neutral’.
4	Dinc & Cooper [83]	2015	UK	Experimental study, ad libitum taste test; mood induction	106	Undergraduate psychology students	60 women	M = 23.915, SD = 6.73, range not reported	Positive	UMACL [36]	No	Ad libitum (milliliters of beverage consumed)	*r* = .43	People consumed more alcohol when in a positive mood compared to a neutral mood.
5	Duif, Thewissen, Wouters, Lechner, & Jacobs [44]	2019	Netherlands	Experience Sampling Method	162	General population	109 women	M = 36.07, SD = 9.27, range = 20–50	Both	PANAS [38]	No	Number of drinks	Within persons: *r* (daily negative affect) = .05; *r* (momentary negative affect) = —.16; *r* (daily positive affect) = .03; *r* (momentary positive affect) = .04. Between persons: *r* (daily negative affect) = .03, *r* (momentary negative affect) = .12; *r* (daily positive affect) = .01; *r* (momentary positive affect) = .01.	Negative affect was not associated with the amount consumed. Higher levels of positive affect were associated with more consumption.
6	Gabel, Noel, Keane & Lisman [84]	1980	USA	Experimental, ad libitum taste test	18	Undergraduate psychology students	Men only	M & SD not reported, range = 18–22	Both	Self-report; basal skin conductance; heart rate	Yes: sexual arousal, fear, neutral	Ad libitum (milliliters of beverage consumed)	*r* (negative) = .11; *r* (positive) = .65	Participants in the sexual arousal condition drank more than in fear or neutral conditions.
7	Gautreau, Sherry, Battista, Goldstein, & Stewart [85]	2015	Canada	Experience Sampling Method	143	Frequent drinkers	105 women	M = 20.78, SD = 3.36, range not reported	Positive	PANAS [38] and mood circumplex [86]	No	Number of drinks	*r* (low arousal) = .03; *r* (high arousal) = .26	High-arousal positive mood was associated with higher number of drinks than low-arousal positive mood.
8	Liu, Wang, Zhan, & Shi [87]	2009	China	Telephone Interviews	37	General population	5 women	M = 31, SD = 8.01, range not reported	Negative	Work stress checklist [88]	Yes—stress	Number of units	*r* = .16	Stress was associated with increased alcohol consumption.
9	Mohr, Arpin & McCabe [89]	2015	USA	Experience Sampling Method	47	Moderate-to-heavy drinkers	23 women	M = 36, SD = 16.98, range not reported	Both	PANAS [38] and mood circumplex [86]	No	Number of drinks	*r* (negative) = .01; *r* (positive) = .04	Affect variability, not mean levels of affect, was associated with elevated consumption.
10	Mohr, Brannan, Wendt, Jacobs, Wright, & Wang [90]	2013	USA	Experience Sampling Method	49	Moderate-to-heavy drinkers	24 women	M = 36, SD = 17.32, range not reported	Both	PANAS [38] and mood circumplex [86]	No	Number of drinks	*r* (positive) = -.16	Participants drank more following increases in both positive and negative mood.
11	Monk, Qureshi, & Heim [32]	2020	UK	Experience Sampling Method	69	General population	42 women	M = 21.47, SD = 4.47, range = 18–36	Both, as a continuum	How would you rate your current mood (on a scale from 0 to 5)?	No	Number of drinks	*r* (negative) = -.31; *r* (positive) = .31	Feeling unhappy prior to the commencement of drinking was a significant predictor of drinking larger quantities of alcohol in the following drinking session.
12	O’Donnel et al. [47]	2019	Australia	Experience Sampling Method	83	General population	63 women	M = 21.42, SD = 3.09, range = 18–30	Both (happy, relaxed, irritated, stressed)	Happy, relaxed, irritated, stressed, on a 6-point scale	Yes (happy, relaxed, irritated, stressed)	Number of drinks	*r* (happy) = .09; *r* (relaxed) = .10; *r* (stressed) = -.09; *r* (irritated) = -.04	Affect was not related to levels of consumption.
13	Peacock, Cash, Bruno, & Ferguson [91]	2015	Australia	Experience Sampling Method	53	General population	22 women	M = 28.2, SD = 11.2, range = 18–60	Both, as a continuum	Visual-analogue Mood Scales [35]	No	Number of drinks	*r* (negative) = -.19; *r* (positive) = .19	Higher positive affect was associated with increased alcohol consumption.
14	Pihl & Yankofsky [92]	1979	Canada	Experimental study, ad libitum taste test; mood induction	40	General population	Men only	M = 20.05, SD not reported, range = 18–27	Negative	MAACL [93]	Yes (depression and anxiety)	Ad libitum (total amount of pure alcohol consumed)	*r* = .28	Less alcohol was consumed by participants who experienced higher depression and anxiety prior to consumption.
15	Richardson, Hoene, & Rigatti [45]	2020	USA	Diary study	222	University students	149 women	M = 20.12, SD = 2.58, range not reported	Both	PANAS [38]	No	Number of drinks	*r* (negative) = .11; *r* (positive) = .17	At low levels of positive affect, individuals higher in self- critical perfectionism reported higher levels of drinking to cope than those lower in self-critical perfectionism. Individuals were also more likely to drink to cope with high negative affect compared to low negative affect.
16	Rohsenow [94]	1982	USA	Experimental study, ad libitum taste test; mood induction	60	Undergraduate students	Men only	M = 23, SD not reported, range = 21–32	Negative	MAACL [93]	Yes (anxiety)	Ad libitum (amount consumed in milliliters, average sip size, total number of sips)	*r* = .31	Those feeling more anxious took fewer sips of alcohol.
17	Simons, Gaher, Oliver, Bush, & Palmer [95]	2005	USA	Experience Sampling Method	56	Moderate drinkers	30 women	M = 21.5, SD = .57, range = 21–23	Both	PANAS [38] and mood circumplex [86]	No	Number of drinks	*r* (negative) = .03; *r* (positive) = .16	Both negative and positive affect were associated with greater consumption volume.
18	Simons, Wills, & Neal [96]	2014	USA	Experience Sampling Method	274	Moderate-to-heavy drinkers	153 women	M = 19.88, SD = 1.37, range = 18–27	Both	PANAS-X [97] and mood circumplex model [86]	No	Number of drinks + transdermal alcohol monitoring	*r* (negative) = .01; *r* (positive) = .07	Daily negative affect was directly associated with higher consumption on drinking days.
19	Stasiewicz & Lisman [98]	1989	USA	Experimental, ad libitum taste test; mood induction	32	Men in risk for future child abuse	Men only	M = 20.6, SD & range not reported	Negative	Blood pressure and heart rate	Yes (aversion, arousal, distress)	Ad libitum (milliliters of beverage consumed)	*r* = .50	Higher aversion, arousal, and distress were associated with higher consumption.
20	Stevenson, Dvorak, Kramer, Peterson, Dunn, Leary, & Pinto [99]	2019	USA	Experience Sampling Method	101	College students	66 women	M = 20.93, SD = 2.89, range = 18–29	Both	PANAS [38]	Yes (depression, anxiety)	Number of drinks	*r* (anxious) = .08; *r* (depressed) = -.01; *r* (positive) = .02	Intending to drink to enhance one’s mood was associated with increased consumption volume.
21	Sutker, Libet, Allain, & Randall [100]	1983	USA	Diary study	32	General population	21 women	Not reported	Negative	MAACL [93]	Yes (anxiety, depression, hostility)	Number of drinks	*r* (anxiety) = -.07, *r* (depression) = -.10; *r* (hostility) = -.02	Negative affect was not associated with consumption.
22	Swendsen, Tennen, Carney, Affleck, Willard, & Hromi [14]	2000	France	Experience Sampling Method	100	Frequent drinkers	55 women	M = 22.9, SD = 4.6, range not reported	Both	Mood Circumplex [86]	Yes (active, peppy, happy, relaxed, quiet, bored, sad, nervous)	Type & Quantity of beverage	*r* (active) = .11; *r* (peppy) = —.05; *r* (happy) = .21; *r* (relaxed) = .12; *r* (quiet) = .32; *r* (bored) = .06; r (sad) = .01; *r* (nervous) = .30	Happy and nervous affective states were associated with increased consumption, feeling quiet was associated with decreased consumption.
23	Wardell, Read, Curtin, & Merrill [101]	2012	USA	Experimental, ad libitum taste test; mood induction	146	College students	67 women	M = 21.45, SD = .73, range = 21–24	Both, as a continuum	Affect Grid [37]	No	Ad libitum (oz of beverage consumed, blood alcohol concentration)	*r* (negative) = -.07; *r* (positive) = .07	Mood was not associated with consumption.
24	Mohr et al. [102]	2005	USA	Experience Sampling Method	122	Undergraduate students	69 women	M = 18.9, SD = 1.16, range not reported	Both	PANAS [38] and mood circumplex [86]	No	Number and volume of drinks	*r* (negative) = .08; *r* (positive) = .07	Both positive and negative mood were positively associated with consumption volume.
25	Hussong, Galloway, & Feagans [103]	2005	USA	Experience Sampling Method	72	College students	36 women	M = 18.10, SD not reported, range not reported	Both	PANAS [38]	Yes (fear, hostility, attentiveness, sadness, shyness)	Number of drinks	*r* (fear) = -.08; *r* (hostility) = .17; *r* (attentiveness) = -.10; *r* (sadness) = .18; *r* (shyness) = -.05	Affect interacted with drinking motives to predict consumption: those high in drinking-to-cope motives drank less on days in which they experienced greater sadness. When experiencing moderate to high levels of fear and shyness, individuals high in drinking-to-cope were more likely to drink. For those low in coping motivations, fear and shyness did not predict daily drinking.
26	Todd, Armeli, Tennen, Carney, & Affleck [104]	2003	USA	Diary study	83	Community sample	44 women	M = 37.2, SD = 6.65, range not reported	Negative	Perceived stress scale [105] and mood circumplex [86]	Yes (anger, boredom, loneliness, nervousness, sadness)	Number, size, and proof of drinks	Study 1. *r* (angry) = .05; *r* (bored) = -.03; *r* (lonely) = -.10; *r* (nervous) = -.06; *r* (sad) = -.05. Study 2. *r* (angry) = 0; *r* (bored) = .02; *r* (lonely) = .05; *r* (nervous) = -.06; *r* (sad) = -.06	Associations between stress/negative affect and drinking outcome variable tend to be near zero for individuals with high drinking-to-cope scores and negative for individuals with low drinking-to-cope scores.
27	Carney, Armeli, Tennen, Affleck, & O’Neil [106]	2000	USA	Diary study	83	Community sample	44 women	M = 37.15, SD = 6.65, range = 26.01–50.76	Negative	Perceived Stress Scale [105]	Yes (stress)	Number, size, and proof of drinks	*r* = .27	Perceived stress was associated with increased consumption.
28	Armeli, Carney, Tennen, Affleck, & O’Neil [107]	2000	USA	Diary study	88	Moderate drinkers	48 women	M = 37.81, SD = 6.92, range not reported	Negative	Modified version of the Assessment of Daily Experience [108]	Yes (stress)	Number of drinks	*r* = -.23	Men who more strongly anticipated positive outcomes or a sense of carelessness from drinking drank relatively more on stressful days compared with low-stress days. Men who anticipated greater impairment from drinking drank relatively less on stressful days. These effects did not hold for women.
29	Dvorak, Pearson, & Day [109]	2014	USA	Experience Sampling Method	74	University students	43 women	M = 21.30, SD = 2.07, range = 18–29	Both	How ___ are you feeling right now (on a scale from 1 to 11)?	No	Number of drinks	*r* (negative men) = -.02; *r* (negative women) = .19; *r* (positive) = -.14	Negative daytime mood was associated with increased consumption, positive daytime mood was not associated with consumption.
30	Thomas, Merrill, von Hofe, & Magid [110]	2014	USA	Experimental, ad libitum taste test; mood induction	112	Frequent drinkers	52 women	M = 27.0, SD = 5.16, range not reported	Negative	Subjective units of distress scale; heart rate; mean arterial pressure; and salivary cortisol.	Yes (stress)	Ad libitum (milliliters of beverage consumed, latency to first sip of beer, average sip size, median latency between sips)	*r* = .10	The stressor did not result in greater consumption of alcohol.
31	Grant, Stewart, & Mohr [111]	2009	Canada	Experience Sampling Method	146	College students	113 women	M, SD, & range not reported	Negative	How ___ did you feel today (on a scale from 0 to 4)?	Yes (depression and anxiety)	Number of drinks	*r* (depressed) = -.07; *r* (anxious) = -.03	Daily depressed mood did not trigger subsequent evening alcohol consumption and daily anxious mood was protective against subsequent evening drinking.
32	Steptoe & Wardle [112]	1999	UK	Diary study	79	Nurses and teachers	45 women	M = 39.75, SD = 9.95, range not reported	Both	POMS [113]	Yes (anxiety)	Number of units	*r* (negative) = .58; *r* (positive) = .58	Consumption of alcohol tended to be greater on days on which participants reported more positive and less anxious mood.
33	Hull & Young [114]	1983	USA	Experimental, ad libitum taste test; mood induction	120	Frequent drinkers	Men only	Over 21, M, SD & range not reported	Negative	MAACL [93]	Yes (anxiety, hostility, depression)	Ad libitum (ounces of beverage consumed)	In high self-conscious subjects: *r* (anxious) = .44; *r* (hostile) = .10, *r* (depressed) = .09. In low self-conscious subjects *r* (anxious) = .09, *r* (hostile) = .10, *r* (depressed) = .09	Negative mood was related to consumption volume in high self-conscious but not low self-conscious participants.
34	O’Hara, Armeli, & Tennen [115]	2014	USA	Diary study	1636	College students	867 women	M = 19.2, SD & range not reported	Both	Items from Mood Circumplex [86] and PANAS-X [97]	Yes (sadness, anxiety)	Number of drinks	*r* (anxiety) = -.06; *r* (anger) = .00; *r* (sadness) = -.20; *r* (positive mood) = .02	Anxiety, anger, and positive mood were positively related to the number of drinks consumed.
35	Todd et al. [116]	2005	USA	Experience Sampling Method	98	Community sample	49 women	M = 43.5, SD & range not reported	Both	Single-item mood measure: How ___ did you feel (on a scale from 0 to 4)?	Yes (peppy, happy, relaxed, bored, sad, nervous, angry, lonely, disappointed)	Number, size, and proof of drinks	*r* (peppy) = .01; *r* (happy) = .05; *r* (relaxed) = .14; *r* (positive mood) = .09; *r* (bored) = .13; *r* (sad) = .07; *r* (nervous) = -.01; *r* (angry) = .05; *r* (lonely) = .06; *r* (disappointed) = .00; *r* (negative moods) = .05	There was no significant association between mood and alcohol consumption.
36	Todd, Armeli, & Tennen [117]	2009	USA	Experience Sampling Method	97	Community sample	48 women	M = 43.5, SD & range not reported	Both	Mood Circumplex [86]	Yes (angry, bored, disappointed, lonely, nervous, sad)	Number, size, and proof of drinks	*r* (angry) = .02; *r* (bored) = .11; *r* (disappointed) = -.01; *r* (lonely) = .04; *r* (nervous) = -.02; *r* (sad) = .07; *r* (negative mood) = .04; *r* (positive mood) = .08	Affective state was not associated with consumption.
37	Collins et al. [118]	1998	USA	Experience Sampling Method	37	Heavy drinkers	15 women	M = 35.92, SD & range not reported	Both	Mood Circumplex [86]	No	Number of drinks	*r* (positive) = .97	Positive but not negative mood predicted excessive drinking.
38	Ehrenberg, Armeli, Howland, & Tennen [119]	2016	USA	Experience Sampling Method	722	College students	391 women	M = 19.24, SD & range not reported	Both	PANAS [38] and mood circumplex [86]	No	Number of drinks	*r* (negative) = .06; *r* (positive) = .01	Consumption level was unrelated to negative affect and positively related to positive affect.
39	Higgins & Marlatt [120]	1975	USA	Experimental, ad libitum taste test	64	Undergraduate psychology students	Men only	M & SD not reported, range = 18–26	Negative	MAACL [93]	Yes (fear of evaluation)	Ad libitum (ounces of beverage consumed and amount of pure alcohol consumed)	*r* = .35	Participants expecting to be evaluated drank significantly more alcohol than low-fear control participants.
40	Holroyd [121]	1978	USA	Experimental, ad libitum taste test	60	Undergraduate students	Men only	Over 18, M, SD, & range not reported	Negative	State Anxiety Scale	Yes (social anxiety)	Ad libitum (numbers of bottles of beer opened, blood alcohol concentration)	*r* = .21	Socially anxious participants and those who received negative social evaluation drank less alcohol.
41	Dvorak, Pearson, Sargent, Stevenson, & Mfon [122]	2016	USA	Experience Sampling Method	74	University students	43 women	M = 21.30, SD & range not reported	Both	PANAS-X [97] and mood circumplex [86]	No	Number of drinks	*r* (negative) = .10; *r* (positive) = .09	Higher positive mood and mood instability were associated with increased consumption.
42	Armeli et al. [123]	2007	USA	Experience Sampling Method	98	Heavy drinkers	49 women	M = 43.5, SD & reported	Both	PANAS [38] and mood circumplex [86]	Yes (stress)	Number of drinks	*r* (negative) = .06	Higher levels of stress and negative affect interacted with individual differences factors to predict increased consumption.
43	Corcoran & Parker [124]	1991	USA	Experimental study, ad libitum taste test; mood induction	69	Undergraduate students	25 women	M, SD, & range not reported	Negative	Personal Evaluation Form	Yes (stress)	Ad libitum (amount of beverage consumed in ounces)	*r* = .17	Stress was not associated with consumption.
44	Kidorf & Lang [125]	1999	USA	Experimental, ad libitum taste test; mood induction; within-subject	84	Undergraduate students	42 women	M = 22.5, SD & range not reported	Negative	MAACL [93]	Yes (stress)	Ad libitum (amount of pure alcohol consumed)	*r* = .08	Stress was positively related to consumption.
45	Tucker, Vuchinich, Sobell, & Maisto [126]	1980	USA	Experimental study, ad libitum taste test; mood induction	43	Heavy social drinkers	Men only	M & SD not reported, range = 18–26	Negative	How anxious did you feel (on a scale from 1 to 7)?	Yes (stress)	Ad libitum (milliliters of alcohol consumed)	*r* = .70	Stress was positively related to consumption.
46	McGrath, Jones, & Field [127]	2016	UK	Experimental study, ad libitum taste test; mood induction	100	Heavy social drinkers	52 women	M = 20.86; SD = 3.93, range not reported	Negative	POMS [112]	Yes (stress)	Ad libitum (milliliters of beverage consumed)	*r* = .21	Stress was positively related to consumption.
47	Magrys & Olmstead [128]	2015	Canada	Experimental study, ad libitum taste test; mood induction	75	Undergraduate students	40 women	M = 20.12, SD & range not reported	Negative	STAI [129]	Yes (stress)	Ad libitum (number of standard drinks consumed, level of intoxication, blood alcohol level)	*r* = .09	Stress was positively related to consumption.
48	Aldridge-Gerry et al. [130]	2011	USA	Experience Sampling Method	365	College students	252 women	M = 20.1, SD & range not reported	Negative	Describe the most stressful event that happened that day and rate on a 5-point scale how stressful it was.	Yes (stress)	Number of drinks	*r* = -.05	Stress was negatively related to consumption.
49	Emery & Simons [131]	2020	USA	Experience Sampling Method	92	Moderate-to-heavy drinkers, undergraduate students	58 women	M = 20.17, SD & range not reported	Both	PANAS-X [97] and mood circumplex [86]	No	Number of drinks	*r* (negative) = .10; *r* (positive) = .15	Positive affect was positively associated with consumption. Negative affect was not associated with consumption.
50	Hamilton, Armeli, & Tennen [132]	2020	USA	Diary study, 2 waves	906	College students	489 women	M = 19.18, SD = 1.26 = > M = 24.56, SD = 1.33, ranges not reported	Both	PANAS [38] and Mood Circumplex [86]	No	Number of standard drinks	Wave 1: *r* (social positive) = .01; *r* (social negative) = -.06; *r* (solitary positive) = -.03; *r* (solitary negative) = .11. Wave 2: *r* (social positive) = .01; *r* (social negative) = .00; *r* (solitary positive) = -.07; *r* (solitary negative) = -.04	Whereas daytime positive affect predicted greater social consumption, it was also related to lower solitary alcohol consumption among college students who were low in state social drinking motives.
51	Tovmasyan, Monk, Bunting, Qureshi, & Heim	Under revision	UK	Experience Sampling Method	79	General population	49 women	M = 29.31, SD = 9.70, range = 20–63	Both	PANAS [38]	Yes (all items from PANAS, [38])	Number of drinks	*r* (between day positive) = —.06; *r* (between day negative) = .01; *r* (within day positive) = -.03; *r* (within day negative) = .03	Being more enthusiastic and less alert was associated with drinking onset, being ashamed was associated with higher number of drinks following drinking onset, feeling strong and interested was associated with decreased drinking volume.
52	Stamates, Linden-Carmichael, Preonas, & Lau-Barraco [46]	2019	USA	Experience Sampling Method	24	Adult drinkers	14 women	M = 23.83, SD = 1.83, range not reported	Both	PANAS [38]	No	Number of standardised drinks	*r* (negative) = .41; *r* (positive) = -.09	Higher negative affect was inversely related to number of drinks consumed.
53	Mohr, Brannan, Mohr, Armeli, & Tennen [133]	2008	USA	Diary study	118	College students	67 women	M = 18.9, SD = 1.16, range not reported	Both	Combination of PANAS [38] and mood circumplex [86]	Yes (angry, sad, bored, nervous, ashamed, hostile, guilty, jittery, dejected)	Number of drinks	Drinking at home: *r* (angry) = .21; *r* (sad) = .04; *r* (bored) = .15; *r* (nervous) = –.05; *r* (ashamed) = .21; *r* (hostile) = .27; *r* (guilty) = .12; *r* (jittery) = .07, *r* (dejected) = .12, *r* (positive mood) = .05. Drinking away: *r* (angry) = .04, *r* (sad) = –.07; *r* (bored) = .05; *r* (nervous) = –.04; *r* (ashamed) = .12; *r* (hostile) = .13; *r* (guilty) = .05; *r* (jittery) = –.02; *r* (dejected) = .01; *r* (positive mood) = .07	Both positive and negative were associated with higher consumption volume.
54	Schroder & Perrine [134]	2007	USA	Interactive Voice Response	173	General population	81 women	M = 42.3, SD = 11.9, range = 21–74	Both	Rate stress, anger, sadness, happiness, quality of the day (the best day I had, the worst day I had) on an 11-point scale	Yes (stress, anger, sadness, happiness)	Number of standard drinks	Between-subject: *r* (stress) = -.08; *r* (anger) = -.05; *r* (sadness) = -.04; *r* (happiness) = -.10; *r* (negative emotions) = -.06; *r* (positive emotions) = -.12. Within-subject: *r* (sadness) = -.02; *r* (anger) = -.01; *r* (stress) = -.03; *r* (happiness) = .10, *r* (negative emotions) = -.02; *r* (positive emotions) = .12	Among women, those with higher average levels of sadness, anger, and stress reported higher levels of alcohol consumption; among men, those with higher negative mood ratings reported significantly less alcohol consumption. When not separated by gender, on both within- and between-participant levels, correlations of mood and drinking did not differ significantly from zero.
55	Waddell, Sher, & Piasecki [135]	2021	USA	Experience Sampling Method	403	General population	202 women	M = 23.3, SD = 7.2, range = 18–70	Negative	5-point Likert scale of distressed and sad	Yes (distressed, sad)	Number of drinks	*r* = -.00	Negative affect did not predict consumption directly but did so through alcohol craving.
56	Larsen, Engels, Granic, & Huizink [136]	2013	Netherlands	Experimental study, ad libitum taste test; mood induction	106	Unversity students	Men only	M = 21.37, SD = 2.32, range = 18–27	Negative	Physiological Arousal Questionnaire (PAQ; [137])	Yes (stress)	Ad libitum (centilitres alcohol consumed	*r* = .14	There was no difference in alcohol consumed between stress and no-stress conditions.
57	Sacco et al. [138]	2015	USA	Telephone interviews	71	Continuing care retirement community	45 women	M = over 80, SD and range not reported	Both	PANAS-S [139]	No	Number of standard drinks	*r* (negative) = -.08; *r* (positive) = .07	No temporal relationship between negative and positive affect and amount consumed.
58	Lindgren et al. [140]	2018		Experimental study, ad libitum taste test; mood induction	149	University students	71 women	M = 21.55, SD = .68, range = 21–25	Both	6-Item Brief Affect Measure [141]	No	Ad libitum (amount in milliliters)	*r* (negative) = .07, *r* (positive) = -.07	Implicit alcohol excite associations were more negatively associated with drinking in negative mood condition and more positively associated with drinking in positive/neutral mood condition.
