# Supplementary material for: Towards an affect intensity regulation hypothesis: Systematic review and meta-analyses of the relationship between affective states and alcohol consumption
Source: PLoS One. 2022 Jan 31;17(1):e0262670. doi: 10.1371/journal.pone.0262670 (PMC8803173; doi:10.1371/journal.pone.0262670)
Supplement: S1 File — (DOCX) [file pone.0262670.s002.docx]

**Supplementary material**

**Unpublished references**

**S1. Reference**

Lenhard, W., Lenhard, A. (Unpublished). Computation of Effect Sizes [Internet].

**S2. Reference**

Tovmasyan, A., Monk, R. L., Bunting, B., Qureshi, A., & Heim, D. (Under revision). Affect And Alcohol Consumption: A Real-Time Study During National Lockdown. – data available on Open Science Framework: <https://osf.io/dhk6j/>
